# Mechanism of oxymatrine in the treatment of cryptosporidiosis through TNF/NF-κB signaling pathway based on network pharmacology and experimental validation

**DOI:** 10.1038/s41598-024-65362-0

**Published:** 2024-06-24

**Authors:** Xiaoning Zhang, Jie Shi, Yilong Lu, Rui Ji, Zhiyu Guan, Fujun Peng, Chunzhen Zhao, Wei Gao, Feng Gao

**Affiliations:** 1 College of Basic Medical Sciences, Shandong Second Medical University, Weifang, 261053 China; 2 College of Pharmacy, Shandong Second Medical University, Weifang, China; 3 College of Traditional Chinese Medicine, Shandong Second Medical University, Weifang, 261053 China

**Keywords:** Cryptosporidiosis, Oxymatrine, Network pharmacology, Protein–protein interaction (PPI) network, Experimental validation, Drug discovery, Microbiology

## Abstract

Cryptosporidiosis is a worldwide zoonotic disease. Oxymatrine, an alkaloid extracted and isolated from the plant bitter ginseng, has been reported to have therapeutic effects on cryptosporidiosis. However, the underlying mechanism of its action remains unclear. In this study, we utilized network pharmacology and experimental validation to investigate the mechanism of oxymatrine in the treatment of cryptosporidiosis. First, the potential targets of drugs and diseases were predicted by TCMSP, Gene Cards, and other databases. Following the intersection of drug-disease targets, the DAVID database was used to implement the enrichment analysis of GO functions and KEGG pathways, and then the network diagram of "intersected target-KEGG" relationship was constructed. Autodock 4.2.6 software was used to carry out the molecular docking of core targets to drug components. Based on the establishment of a mouse model of cryptosporidiosis, the validity of the targets in the TNF/NF-κB signaling pathway was confirmed using Western blot analysis and Quantitative Rea-ltime-PCR. A total of 41 intersectional targets of oxymatrine and *Cryptosporidium* were generated from the results, and five core targets were screened out by network analysis, including RELA, AKT1, ESR1, TNF, and CASP3. The enrichment analysis showed that oxymatrine could regulate multiple gene targets, mediate TNF, Apoptpsis, IL-17, NF-κB and other signaling pathways. Molecular docking experiments revealed that oxymatrine was tightly bound to core targets with stable conformation. Furthermore, we found through animal experiments that oxymatrine could regulate the mRNA and protein expression of IL-6, NF-κB, and TNF-α in the intestinal tissues of post-infected mice through the TNF/NF-κB signaling pathway. Therefore, it can be concluded that oxymatrine can regulate the inflammatory factors TNF-α, NF-κB, and IL-6 through the TNF/NF-κB signaling pathway for the treatment of cryptosporidiosis. This prediction has also been validated by network pharmacology and animal experiments.

## Introduction

Cryptosporidiosis(CPS) is a zoonotic infection caused by *Cryptosporidium parvum*, a zoonotic protozoan parasite that causes diarrhea and other diseases in humans^1^. *Cryptosporidium* is a parasite that lives primarily on the surface of the epithelial cells of the host gastrointestinal tract and is transmitted by fecal–oral transmission, mainly in the form of oocysts, through waterborne and droplet transmission. It is increasingly recognized as one of the major causes of moderate to severe diarrhea in developing countries. Of the 20 *Cryptosporidium* species and genotypes that have been reported in humans, *Cryptosporidium humanum* and *Cryptosporidium parvum* are the major causative agents^2^. In previous case reports, children were the main source of infection. In 2016, *Cryptosporidium* infection was reported to be the fifth leading cause of diarrhea in children under 5 years of age^3^. Research has shown that *Cryptosporidium* infects mainly the hind jejunum and ileum of the host, and then spreads to the colon and rectum, leading to apoptosis, intestinal villi atrophy, shortening and fusion, and trap hyperplasia^4^.

Numerous drug studies have been conducted to explore the treatment of cryptosporidiosis, including statins^5^ and various antibiotics^6^. Despite these efforts, the optimal treatment for cryptosporidiosis has not yet been identified. In contrast, herbal medicines have emerged as an important research area for treating cryptosporidiosis. They have the advantages of multi-target, multi-component, and multi-action pathways, and show low side effects and less susceptibility to drug resistance. Studies have suggested that herbal components such as allicin^7–9^, sophora flavescens^10^, and astragalus^11^ are significantly effective in treating cryptosporidiosis.

Oxymatrine (OMT) is one of the main components in the traditional Chinese medicine Sophora flavescens, which has the function of inhibiting apoptosis and proliferation and plays a role in the therapeutic efficacy in treating various conditions, including anti-tumor^12,13^, anti-fibrotic^14^, anti-viral^15^, and anti-inflammatory^16^ diseases. It has been shown that oxymatrine can repair *Cryptosporidium*-infected intestinal mucosa by regulating TLR in mouse intestinal mucosa tissue^17^. Therefore, investigating the mechanism of action of oxymatrine in *Cryptosporidium* infection is of particular importance. In this paper, we proposed a multi-target and multi-pathway research approach based on network pharmacology and molecular docking technology to explore oxymatrine's mechanism in cryptosporidiosis, which provides guidance for the research and the application of oxymatrine in the treatment of cryptosporidiosis.

## Materials and methods

### Network pharmacology methods

#### Obtaining gene targets for oxymatrine

According to the search method of Ye et al.^18^, the targets of oxymatrine were obtained from the Traditional Chinese Medicine Systems Pharmacology Database (TCMSP, https://tcmsp-e.com/tcmsp.php) and PharmMapper database (http://www.lilab-ecust.cn/pharmmapper/) using the compound name “oxymatrine”. Then the main components and related targets of oxymatrine were extracted through literature search. By transforming the relevant targets using the Uniprot database (https://www.uniprot.org/), the gene targets of oxymatrine were obtained from three different databases after gene naming and merging and de-duplication.

#### Obtaining gene targets for cryptosporidiosis

*Cryptosporidium* disease targets were obtained by searching the Gene Cards Database, Online Mendelian Inheritance in Man (OMIM, https://www.genecards.org/), Therapeutic Target Database (TTD, https://db.idrblab.net/ttd/), Comparative Toxicogenomics Database (CTD, http://ctdbase.org/) and related literature with the search term “Cryptosporidiosis, Cryptosporidiosis, *Cryptosporidium parvum*, *Cryptosporidium enteritis*, *Cryptosporidium*, *Cryptosporidium hominis*, Foodborne *Cryptosporidium*, *Cryptosporidium* infection, Zoonotic cryptosporidiosis, *Cryptosporidium parvum* oocysts”, and were then merged and de-duplicated.

#### Protein–protein interaction data

In the Venny online platform, the gene targets of oxymatrine intersect with the gene targets of cryptosporidiosis. The intersected targets were introduced in the Search Tool for the Retrieval of Interaction Gene/Proteins Database (STRING, http://string.embl.de/). The “human” species was selected, and “Multiple proteins” was chosen. Subsequently, the free targets were removed, and the file was imported into Cytoscape 3.9.0 for visualization, generating protein–protein interaction (PPI) network and plotting a histogram of target degree values.

#### Analysis of biological functions and pathways

We entered the drug-disease intersectional target in the Database for Annotation Visualization and Integrated Discovery (DAVID, https://david.ncifcrf.gov/) and selected it as “Homo sapiens”. By analyzing the GO molecular function and KEGG pathway enrichment of the target, the potential pathway and function of oxymatrine for the treatment of cryptosporidiosis were obtained.

#### Construction of intersectional target-KEGG network

Cytoscape 3.9.0 software was used to build the Intersectional Target-KEGG network. Then, the collected results of the intersectional target and KEGG enrichment analysis were input into the software for ranking and analyzing the network nodes by their degree values.

#### Molecular docking analysis of drugs and core protein receptors

In the intersectional target-KEGG network analysis, the top 5 key targets were selected for molecular docking with oxymatrine by degree value ranking. The PDB files of the protein structures of the 5 key targets were downloaded from the Protein Data Bank (PDB, https://www.rcsb.org/). Then, we searched the PubChem database (https://pubchem.ncbi.nlm.nih.gov/) with the keyword “oxymatrine”, downloaded the SDF files of their 2D structures, and then converted them into mol2 structure files by Openbable software. Molecular docking was performed by AutoDuck software, and the docking results were visualized by Pymol.

### Experimental assessment

#### Reagents and chemicals

RIPA Lysis Buffer (R0020) and BCA kit (PC0020) were purchased from Solarbio (China). SDS PAGE Gel preparation Kit (P0012AC) was purchased from Beyotime (China). RNA extraction kit (AG21023), reverse transcription kit (AG11728), and SYBR Green Premix Pro Taq HS qPCR Kit (AG11701) were purchased from Accurate Biotechnology (Hunan, China) Co., Ltd. The antibodies used are as follows: TNF-α (#3707) was obtained from Cell Signaling Technology. IL-6 (AB259341) was obtained from Abcam. β-actin(AB0035) and Goat Anti-Rabbit lgG(H + L) HRP(AB0101) were obtained from Abways technology.

#### Animal and *cryptosporidium* oocysts

This study was approved by the Animal Ethics Committee of Weifang Medical University, with the ethical approval number 2019SDL059. We confirm that all experiments were performed in accordance with relevant named guidelines and regulations.Throughout the experimental process, all methods are reported in accordance with ARRIVE guidelines.

Thirty Specific Pathogen-Free (SPF) healthy female BALB/c mice, four weeks old, were purchased from Jinan Pengyue Laboratory Animal Breeding Co., Ltd, with license approval number SCXK (lu)2022 0006. *Cryptosporidium* oocysts (subtype:IIdA19G1) were generously donated by Prof. Zhang Longxian of Henan Agricultural University.

#### Establishment of animal models and grouping

Thirty 4-week-old healthy female BALB/c mice were randomly divided into 3 groups (control, CPS model control, and OMT treatment), with 10 mice in each group. The mice in the infected group were orally infected with *Cryptosporidium* oocysts at a concentration of 1 × 10^5^ per mouse. In the infected group, we added 5 mg/L dexamethasone sodium phosphate injection into their drinking water for immune function suppression before infection for a duration of 7 days, and immediately discontinued once successfully infected Additionally, in the infected group, we added 40 mg/L of gentamicin sulfate injection into the drinking water of the infected group before infection for intestinal antibacterial for a duration of 7 days, and immediately discontinued after the mice were successfully infected. The control group received a normal diet and water. On the second day of infection, fecal smear was taken to find oocysts, while simultaneously observing the body changes of mice. If most of the mice appear hypokinetic, anorexia, emaciation, fluffy body hair and other phenomena, and some of them occur diarrhea, fecal smear to find oocysts.

On the 7^th^ day of infection, a successful mouse model of *Cryptosporidium* intestinal infection was established. The mice in the treatment group received oxymatrine through gavage at the dosage of 50 mg/kg (0.1 ml/mouse) for 2 weeks, initiated after successful modeling. Meanwhile, the mice in the infected group were dissected and euthanized at the end of the 2nd week post-infection. Following 2-week drug treatment, the mice in the treatment group were also dissected and euthanized for subsequent analysis.

#### Quantitative real-time-PCR analysis (qRT‑PCR)

The jejunum tissue of a mouse was homogenized, and the total RNA concentration was determined through the use of an RNA Extraction Kit. The extracted RNA underwent reverse transcription into cDNA using the reverse transcription kit, following the instructions provided in the manual. Quantitative Real-time-PCR was then used to determine the relative expression of TNF-α, NF-κB, and IL-6 mRNA. Reaction conditions were: predenaturation at 95 ℃ for 30 s, denaturation at 95 ℃ for 5 s, and annealing at 60 ℃ for 30 s, a total of 50 cycles. The melting curve was set as default. The relative mRNA expression was calculated by 2^-ΔΔCt^. The primers are listed in Table 1.

**Table 1:** Sequences of the primers used for qRT-PCR.

Accession numbers	Gene	Primers
NM_013693	TNF-α	F:ACTCCAGGCGGTGCCTATGT
		R:GTGAGGGTCTGGGCCATAGAA
NM_009045	NF-κB	F:TGCATATAGCGGCCGGAAG
		R:CCCAAGAGTCGTCCAGGTCATAG
NM_031168	IL-6	F:CCACTTCACAAGTCGGAGGCTTA
		R:CCAGTTTGGTAGCATCCATCATTTC
NM_001289726	GAPDH	F:GCACCGTCAAGGCTGAGAAC
		R:TGGTGAAGACGCCAGTGGA

#### Western blotting

Mouse jejunal tissue samples were collected, and cell lysate, phosphatase, and protease inhibitors were added to allow adequate lysis. After centrifugation, the supernatant was collected. The protein concentrations were measured with the BCA kit. Proteins were separated through SDS-PAGE gel, transferred to the membrane, and blocked with 5% BSA-TBST for 1 h. Membranes were cut prior to hybridisation with antibodies: TNF-α(1:1000), IL-6(1:1000),β-actin(1:5000) and left to incubate at 4 °C overnight. After washing the membrane, we added the secondary antibody (1:5000). The membranes were washed 3 times with tris buffered saline + Tween (TBST) for 10 min. The experiment utilized an ECL chemiluminescent reagent to develop protein bands, which were then captured and analyzed using the ImageJ software for quantification.

### Statistical analysis

Statistical analysis of experimental data was performed using GraphPad Prism v.9.0.0 software, with expressing data as mean ± SD. The difference between groups was revealed according to t-test. All data were collected from at least three independent experiments. *P* < 0.05 is considered statistically significant.

## Results

### Results of network pharmacology analysis

#### Gene target of oxymatrine

The genes were obtained from the TCMSP database and Pharm Mapper database, and the gene names were transformed by the Uniprot database to obtain the gene targets of oxymatrine. Then, the main components and related targets of oxymatrine were extracted through related literature for supplementation, and the related targets in the three databases were combined and de-duplicated, obtaining 189 gene targets of oxymatrine.

#### Gene targets of cryptosporidiosis

By conducting separate searches from Gene Cards, OMIM, TTD, and CTD databases, and supplementing with relevant literature, a total of 1378 relative targets were obtained, which were then combined and de-weighted.

#### Intersectional targets of diseases and drugs and building protein interaction networks

**Figure 1:**
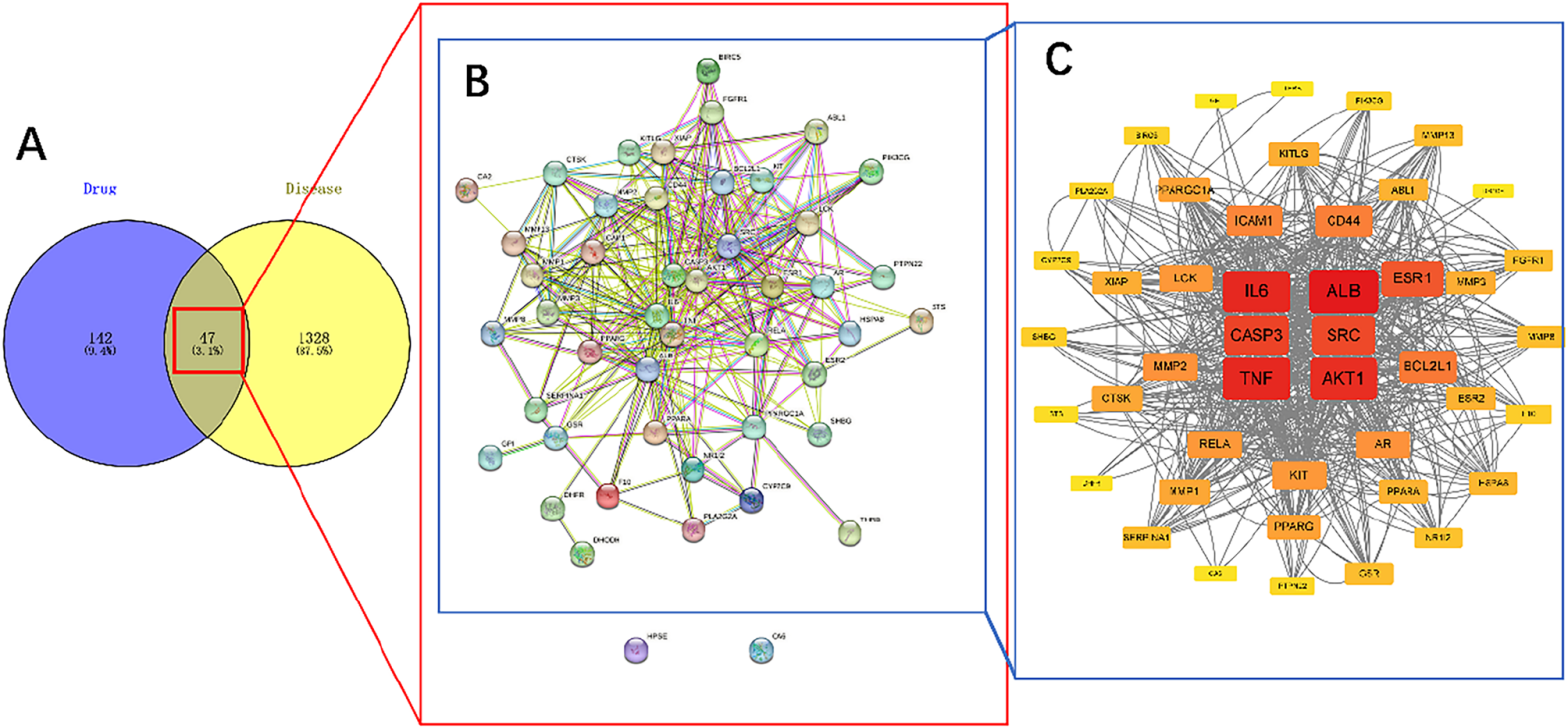
PPI network for the treatment of cryptosporidiosis with oxymatrine. A: Venn diagram—communicative targets, with numbers indicating the count of targets. B: PPI network constructed by STRING. C: PPI network constructed by Cytoscape. The nodes represent targets, and the edges represent the interactions between the targets. As the degrees increase, the color of the nodes changes from yellow to red, and the sizes of the nodes increase.

**Figure 2:**
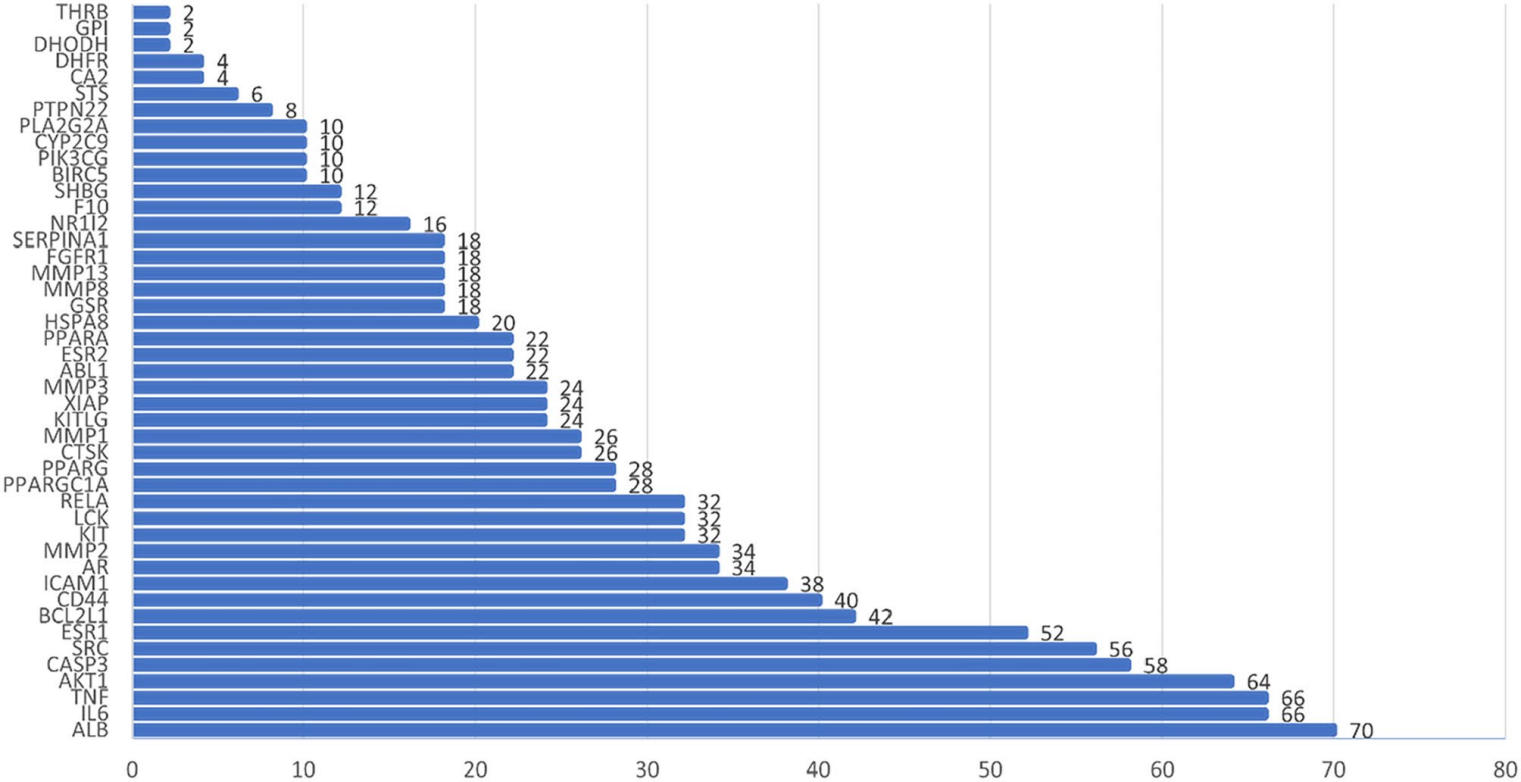
Degree value of the intersectional target.

The obtained 189 gene targets of oxymatrine and 1378 gene targets of cryptosporidiosis were imported into Venny online platform to construct Venn diagrams, and a total of 47 intersectional targets were obtained. The intersectional targets were imported into the STRING database to perform network analysis. The isolated targets were removed and the network was visualized using Cytoscape 3.9.0 (Fig. 1). By ranking the degree values (Fig. 2), it can be seen that ALB (degree = 70), IL6 (degree = 66), TNF (degree = 66), AKT1 (degree = 64), CASP3 (degree = 58), and SRC (degree = 56) interacted more frequently, indicating that these six targets play a stronger role in the treatment of cryptosporidiosis with oxymatrine.

#### Analysis of biological functions and pathways

The intersectional targets of diseases and drugs were imported into the DAVID database to obtain the enrichment results of GO actions and KEGG pathways. A total of 84 KEGG pathways, 29 cellular components (CC), 241 biological processes (BP), and 54 molecular functions (MF) of GO functions were obtained.

**Figure 3:**
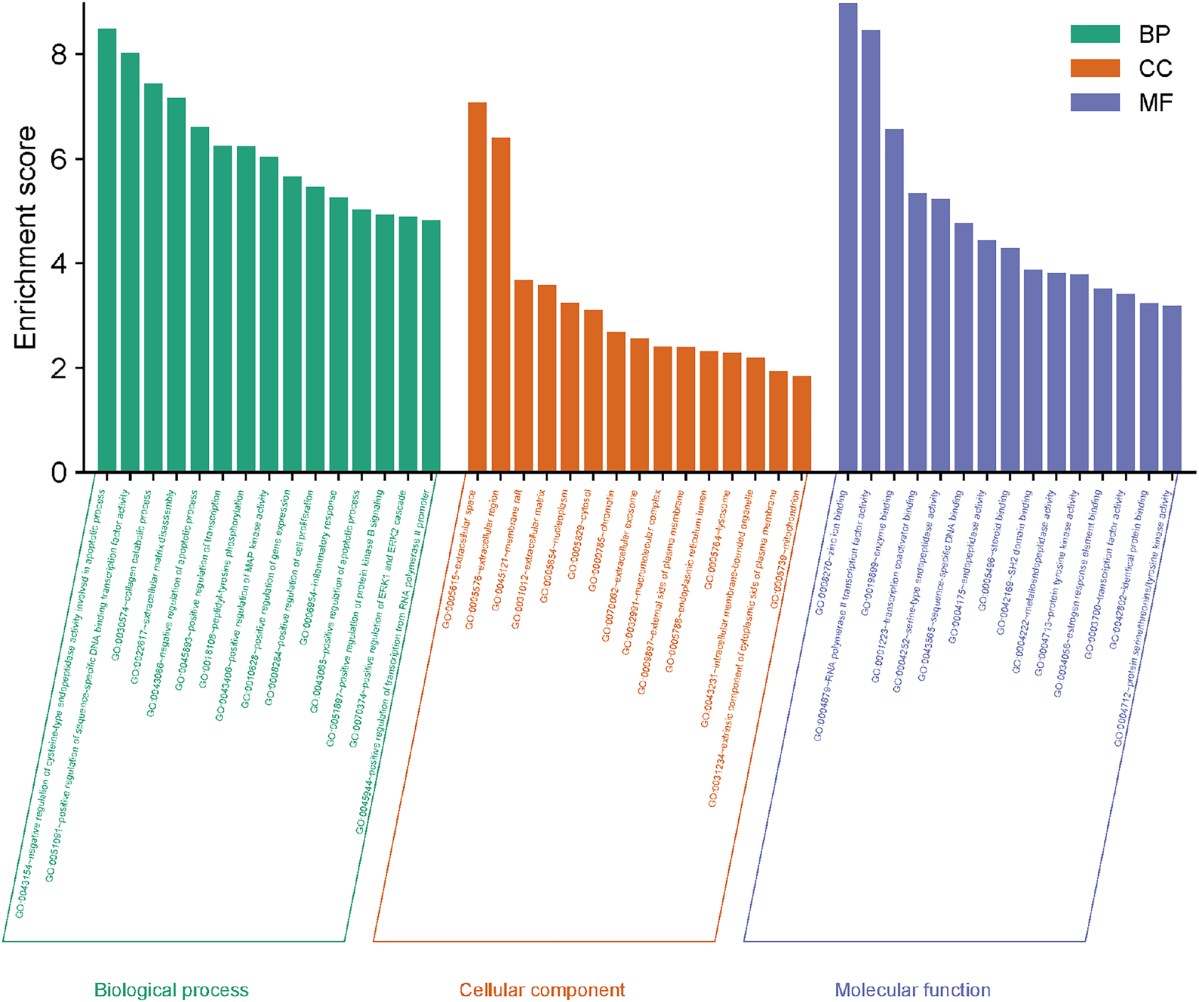
Top 15 GO enrichment analyses. The y-axis represents the enrichment gene count, and the x-axis represents terms related to BP, CC, or MF. Green is BP, Orange is CC, and Purple is MF.

The top 15 GO functional entries of P-value were selected separately for visualization (Fig. 3). It can be seen that the cellular components mainly include extracellular space, extracellular region, membrane raft, extracellular matrix, nucleoplasm, etc. The biological processes mainly include negative regulation of cysteine-type endopeptidase activity involved in the apoptotic process, positive regulation of sequence-specific DNA binding transcription factor activity, collagen catabolic process, etc. The molecular functions include zinc ion binding, RNA polymerase II transcription factor activity, enzyme binding, transcription coactivator binding, etc. The results indicate that oxymatrine could be used to treat cryptosporidiosis through multiple GO functions.

**Figure 4:**
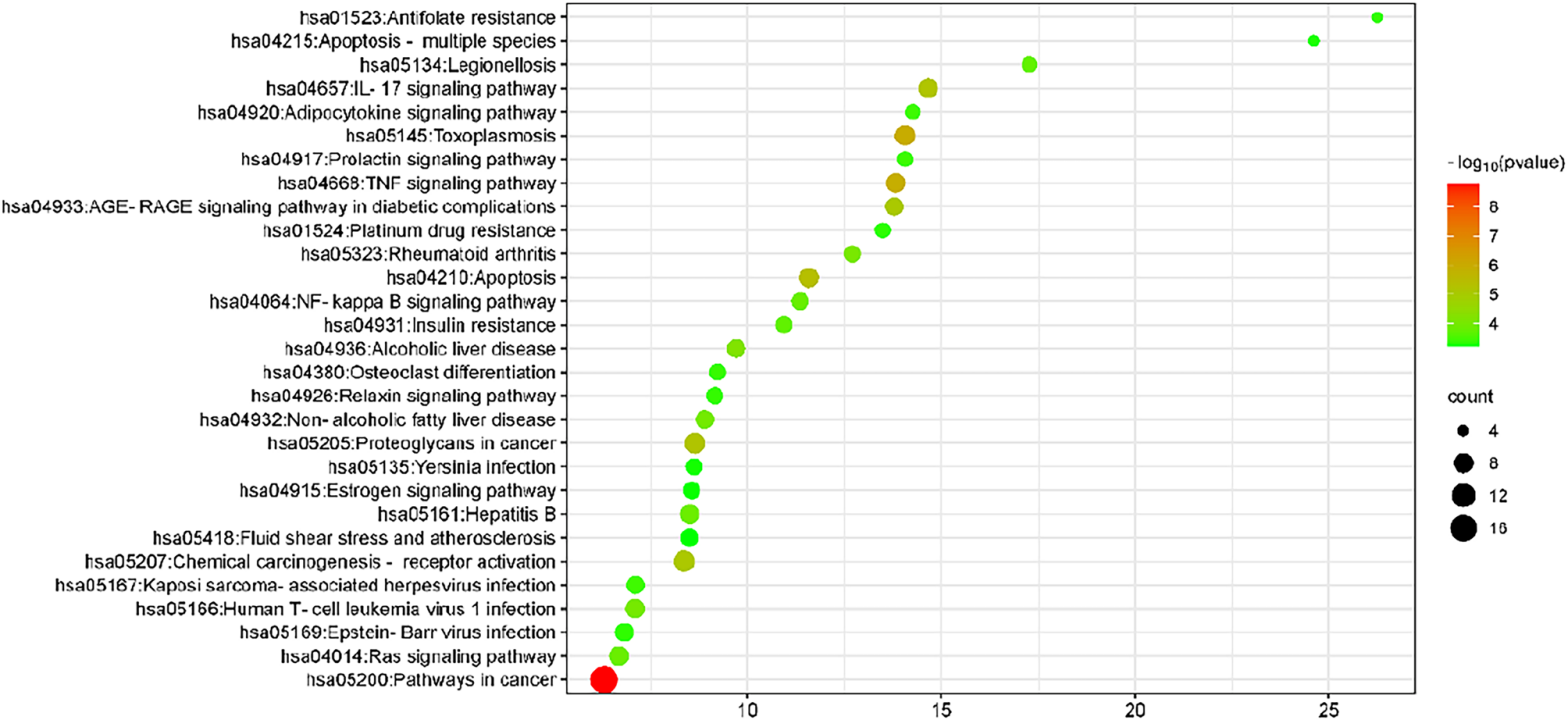
Top 30 KEGG pathway enrichment analyses. The x-axis represents the enrichment gene ratio, and the y-axis represents terms related to the KEGG pathway. The bubble size indicates the enrichment gene count.

**Figure 5:**
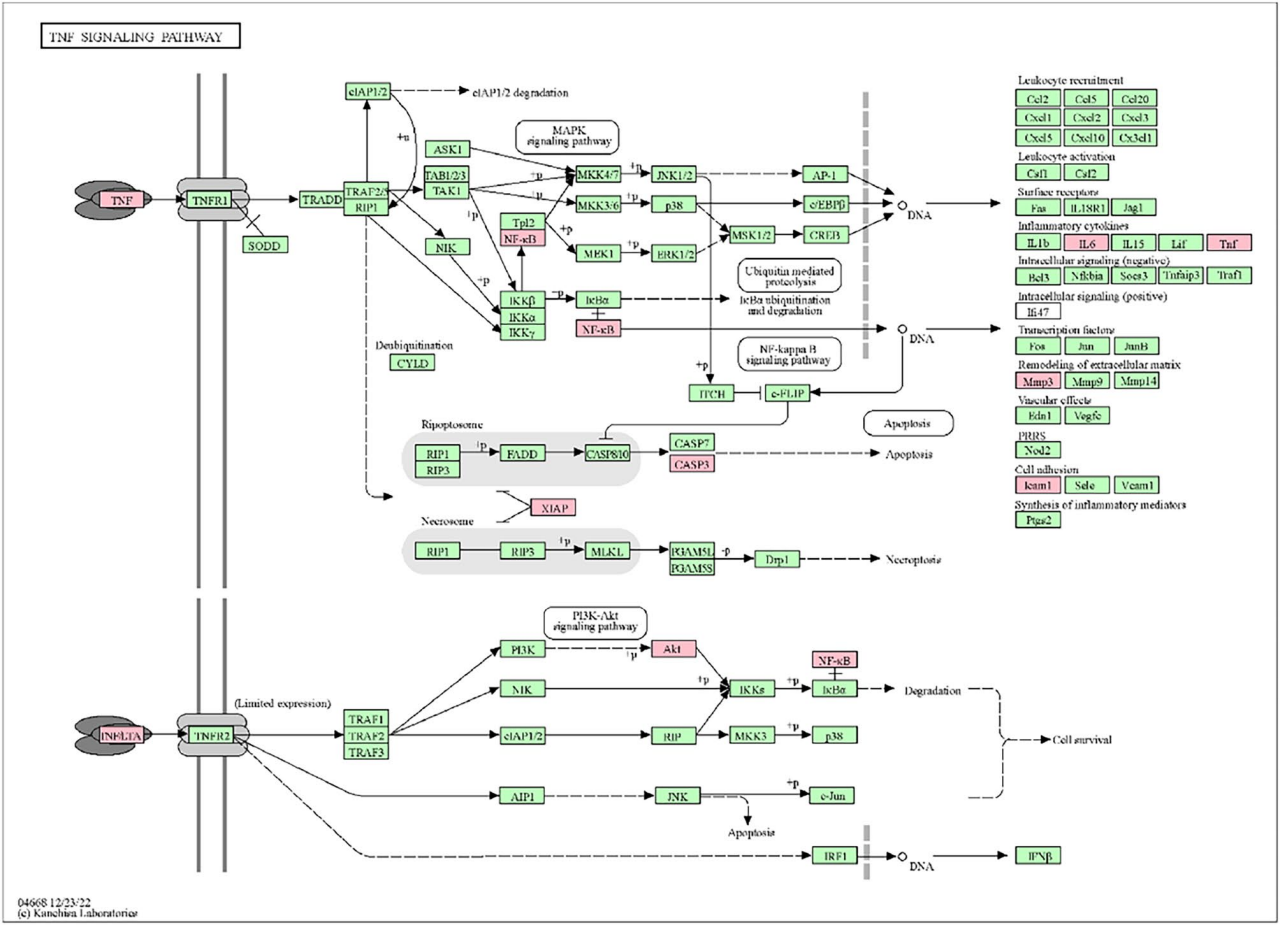
TNF signaling pathways. The red rectangle nodes represent the most significant genes or biological pathways associated with oxymatrine pharmacological actions (KEGG Mapper Color).

The visualization in Fig. 4 presents the top 30 KEGG pathway entries. Noteworthy pathways of oxymatrine action on cryptosporidiosis include the TNF signaling pathway, Apoptosis, IL-17 signaling pathway, NF-κB signaling pathway, etc. It suggests that these pathways may be closely related to the treatment of cryptosporidiosis. Particularly, the TNF signaling pathway is highly ranked and may play an important role in treating cryptosporidiosis with oxymatrine. Figure 5 shows the TNF signaling pathway, and the red rectangular nodes represent genes associated with the pharmacological effects of oxymatrine.

#### Intersectional target-KEGG network

**Figure 6:**
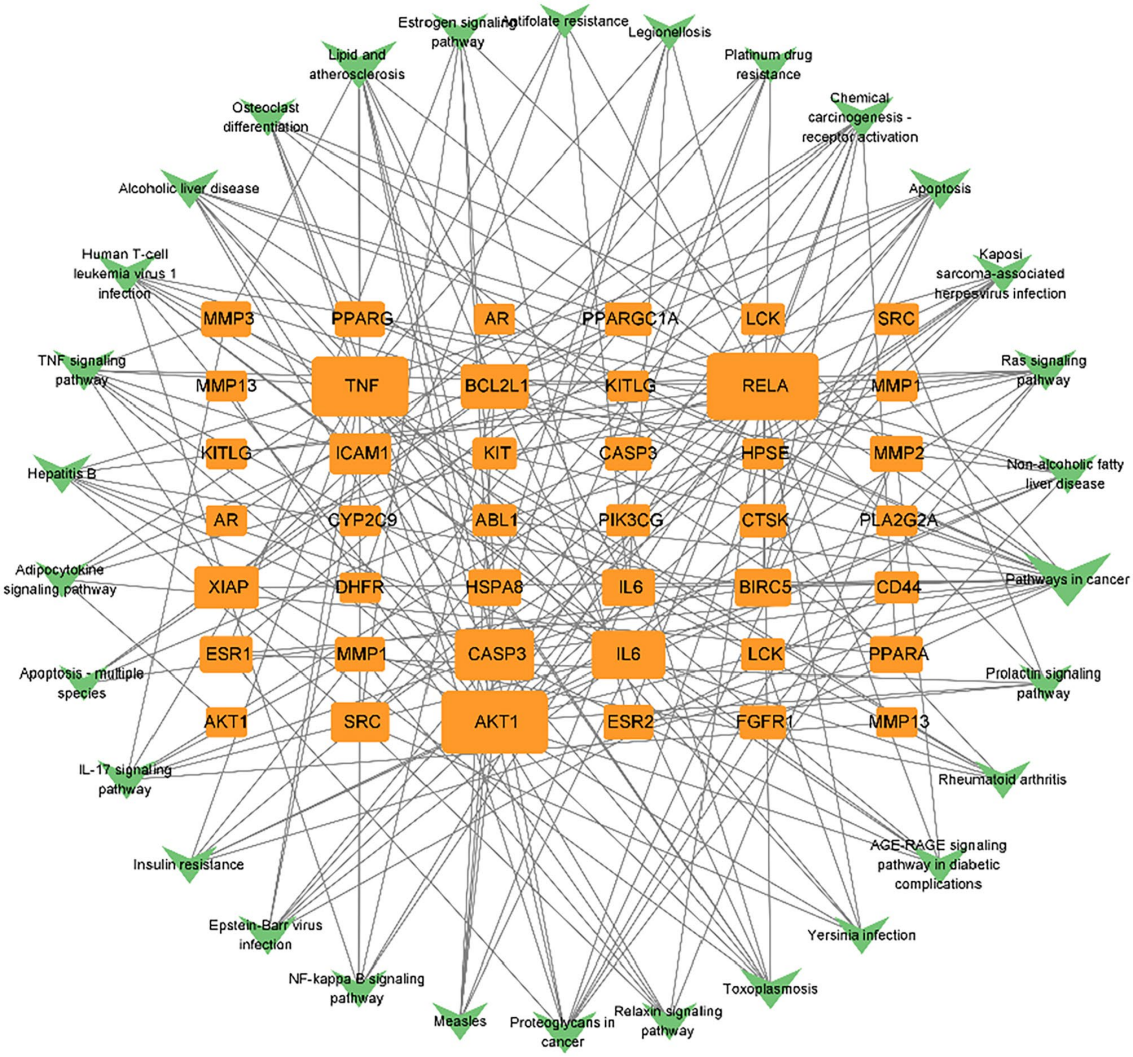
Target-pathway network of oxymatrine in the treatment of cryptosporidiosis. The V shapes represent intersectional targets and the square represent KEGG pathways.

Cytoscape 3.9.0 software was used to analyze the intersectional gene-KEGG network, involving 47 intersectional targets and the top 30 KEGG signaling pathways (Fig. 6). The connecting lines between each node indicate a potential connection between them. A higher number of edges between nodes corresponds to increased importance of those nodes, reflected in their higher degree values. This elevated degree value suggests the involvement of multiple targets and pathways in the treatment of cryptosporidiosis with oxymatrine. Then, the top 5 genes by degree value were found to be RELA (degree value = 25), AKT1 (degree value = 23), TNF (degree value = 20), CASP3 (degree value = 14), and IL-6 (degree value = 12). Among these, the top 10 signaling pathways are Pathways in cancer, Lipid and atherosclerosis, Proteoglycans in cancer, Chemical carcinogenesis—receptor activation, Toxoplasmosis, TNF signaling pathway, Apoptosis, Human T-cell leukemia virus 1 infection, Ras signaling pathway, and IL-17 signaling pathway. This reaffirms that the TNF signaling pathway is particularly important in treating cryptosporidiosis with oxymatrine.

#### Molecular docking

The top 5 core targets, i.e., RELA, AKT1, IL-6, TNF, and CASP3, were selected in the intersectional target-KEGG network analysis for molecular docking with oxymatrine, and the binding energies were derived (Table. 2). The binding energy less than 0 indicated that the two could dock freely and stably. The smaller the binding energy, the stronger the molecular binding ability, and the more stable the confirmation. The target with the lowest binding energy was AKT1 with a value of − 8.63 kJ/mol, followed by RELA, IL-6, CASP3, and TNF with a binding energy of − 8.41 kJ/mol, − 7.43 kJ/mol, − 6.63 kJ/mol, and − 6.19 kJ/mol, respectively.

**Table 2:** Binding energy of molecular docking of the core target with oxymatrine.

Core target	Drug ingredients	Binding energy(kJ/mol)
RELA	Oxymatrine	− 8.41
AKT1	Oxymatrine	− 8.63
IL-6	Oxymatrine	− 7.43
TNF	Oxymatrine	− 6.19
CASP3	Oxymatrine	− 6.63

**Figure 7:**
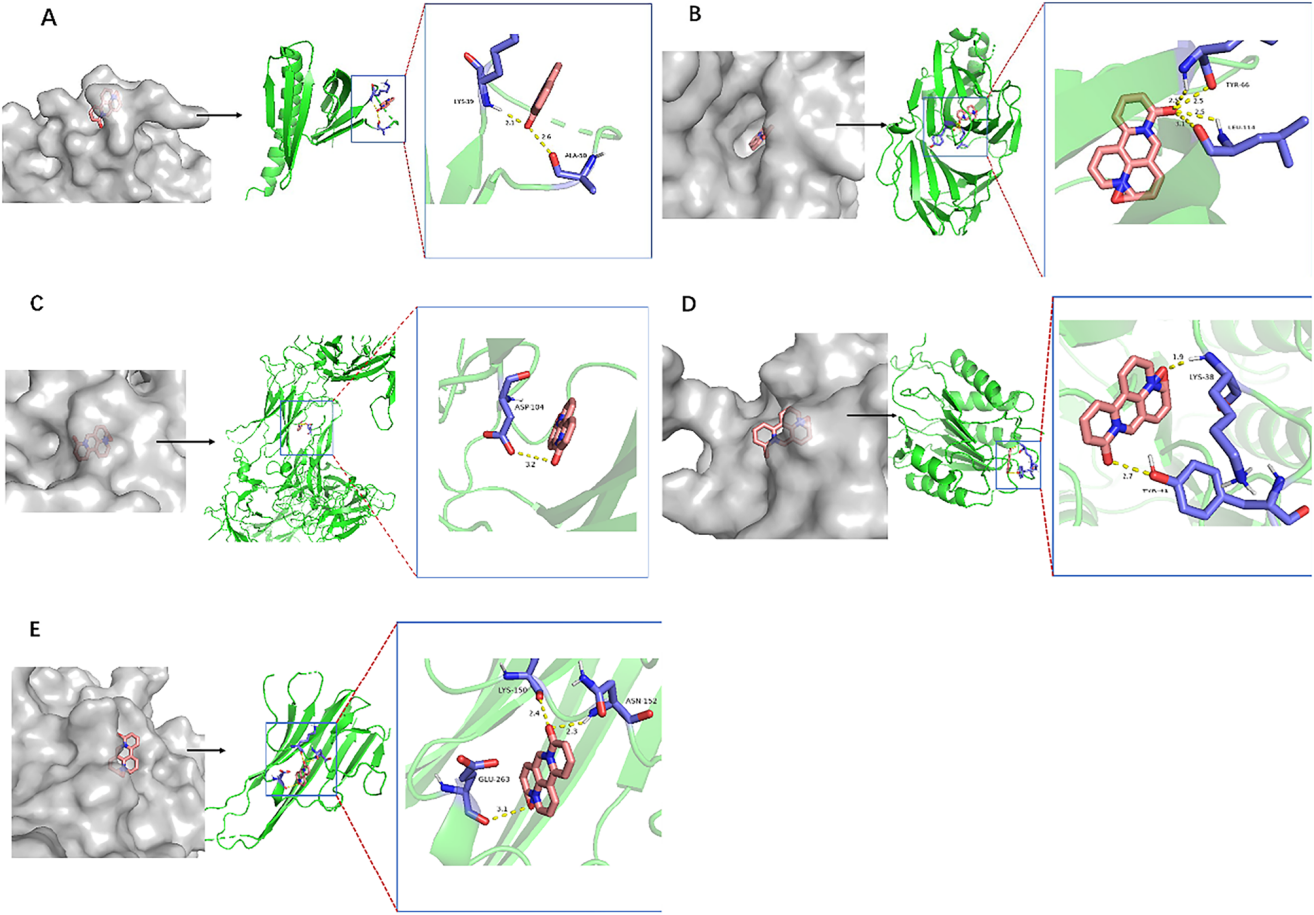
Docking models of core compounds and core targets. The left side of each picture shows a model of an active pocket of a compound encapsulated in a receptor protein. The right side shows the 3D interaction diagrams of the compounds and the targets. The compounds are represented by sticks. The targets are shown in the ribbon model, yellow dashed lines represent the hydrogen bonds, and binding site residues are displayed in lines and labeled with amino acid codes. A: AKT1 and oxymatrine; B: RELA and oxymatrine; C: IL-6 and oxymatrine; D: CASP3 and oxymatrine; E: TNF and oxymatrine.

As shown in Fig. 7, AKT1 combines with LSY-39 and ALA-50 to form hydrogen bonds with bond lengths of 2.1 and 2.6, respectively; RELA combines with TYR-66 and LEU-114 to form hydrogen bonds with bond lengths of 2.5 and 3.1, respectively; IL-6 combines with ASP-104 to form hydrogen bonds with bond lengths of 3.2, respectively; CASP3 combines with LYS-38 and TYR-41 to form hydrogen bonds with bond lengths of 1.9 and 2.7, respectively; TNF combines with LYS-150, ASN-152, and GLU-263 to form hydrogen bonds with bond lengths of 2.4, 2.3 and 3.1, respectively. All the above compounds are well encapsulated in the active pocket of the receptor protein, which also fully verifies the binding mode of the gene target and the drug.

### Experimental assessment

#### Relative mRNA expression of TNF-α, NF-κB, and IL-6 in mouse intestinal mucosal tissues

**Figure 8:**
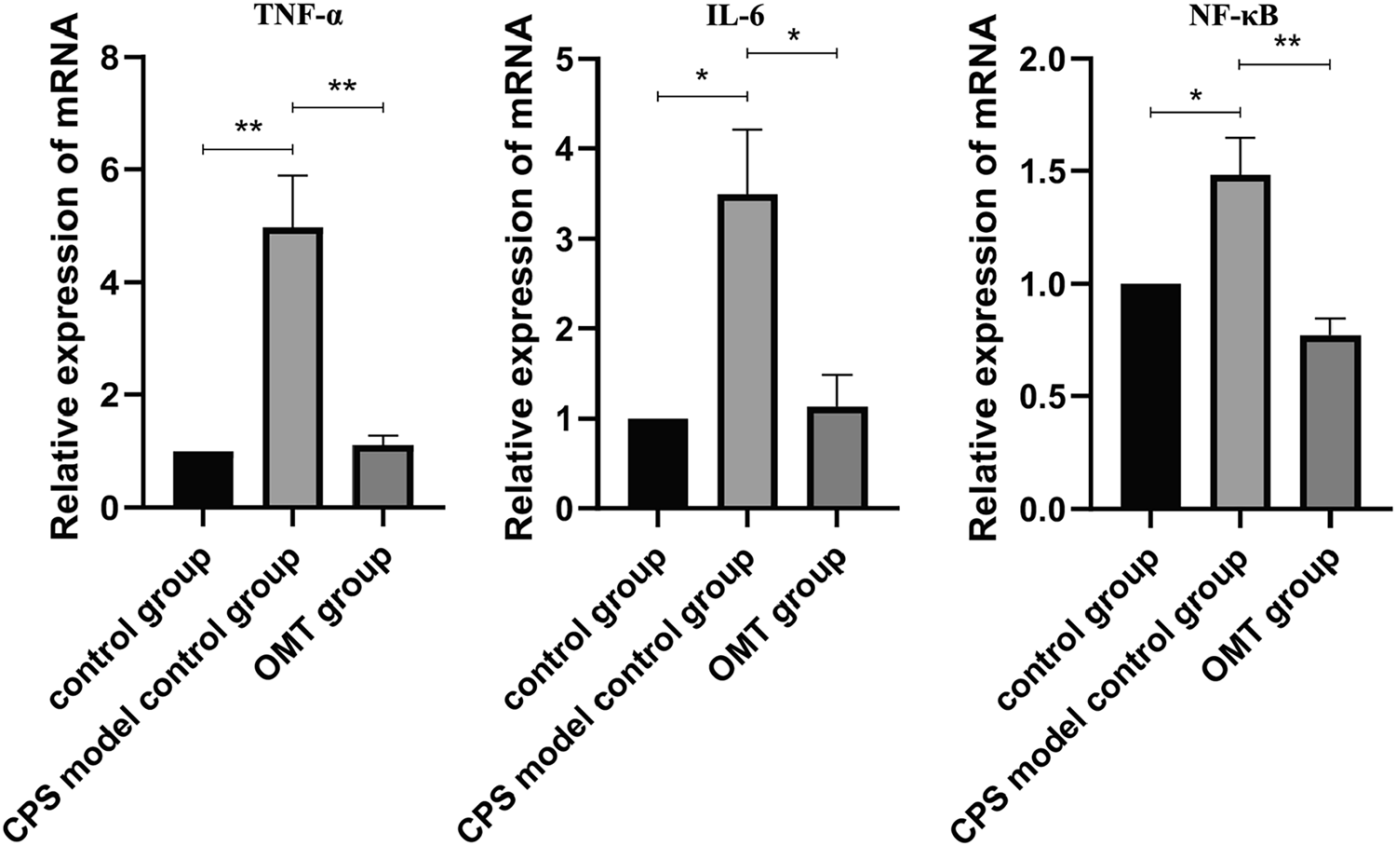
qRT-PCR analysis of TNF-α, IL-6 targets relative mRNA expression.* *p* < 0.05, ** *p* < 0.01, *** *p* < 0.001, ns, No significant difference.

The results of the qRT-PCR test revealed that the relative mRNA expression of TNF-α, NF-κB, and IL-6 increased in the intestinal mucosal tissues of mice infected with *Cryptosporidium*. The expression levels of TNF-α (t = 4.98, *p* < 0.001), NF-κB(t = 1.48,* p* < 0.05) and IL-6 (t = 3.49, *p* < 0.05) were significantly and statistically higher in the infected mice compared to the normal control group. However, the relative mRNA expression of TNF-α, NF-κB, and IL-6 was significantly reduced in mice treated with oxymatrine. Compared with the normal control group, the expression levels of TNF-α (t = 1.12, *p* < 0.001), NF-κB(t = 0.77, *p* < 0.001) and IL-6 (t = 1.13, *p* < 0.05) were significantly and statistically lower in the treated group of mice (Fig. 8). The results showed that oxymatrine can mediate the host inflammatory response caused by *Cryptosporidium* by regulating TNF-α, NF-κB and IL-6, suggesting an obvious anti-inflammatory effect.

#### Relative protein expression of TNF-α and IL-6 in mouse intestinal mucosal tissues

**Figure 9:**
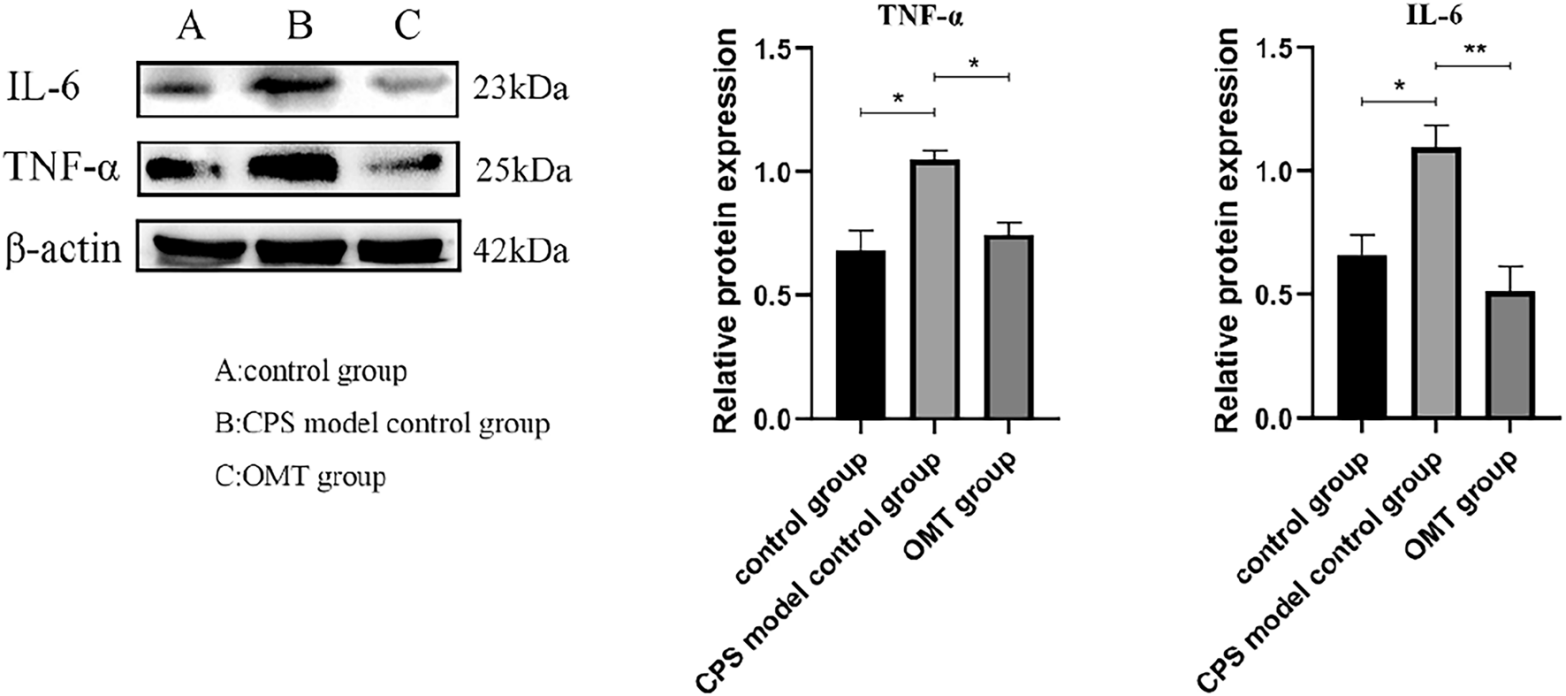
Western-blotting analysis of protein expressions of TNF-α and IL-6, with β-actin employed as an internal control. * p < 0.05, ** p < 0.01, ns, no significant difference.

Relative protein expression of TNF-α and IL-6 in mouse intestinal mucosal tissues was assessed through Western blotting. In mice infected with *Cryptosporidium*, there was an observable upward trend in the expression of TNF-α and IL-6 proteins. The expression levels of TNF-α (t = 1.05, *p* < 0.05) and IL-6 (t = 1.10, *p* < 0.05) were significantly and statistically higher in the infected mice compared to the normal control group. However, in the group of mice that treated with oxymatrine, the relative expression of TNF-α and IL-6 proteins was significantly reduced. The expression level of TNF-α (t = 0.75, *p* < 0.05) and IL-6 (t = 0.51, *p* < 0.01) was significantly and statistically lower in the treated mice compared to the infected mice (Fig. 9).

## Discussion

At present, the main symptom of cryptosporidiosis is diarrhea, which is especially fatal to patients with AIDS. However, there are no specific drugs for the treatment of cryptosporidiosis. Therefore, the research of drugs for cryptosporidiosis has been a research hotspot. In recent years, it has been found that Sophora flavescens has preventive, antibacterial, anthelmintic, anti-inflammation, enhancement of cellular immunity, protection of intestinal mucosa, etc., and has a potential therapeutic effect on cryptosporidiosis^19–21^. In addition, oxymatrine, as one of the main components in Sophora flavescens, has research value for the treatment of cryptosporidiosis. Oxymatrine is highly abundant and easily available in plants such as bitter ginseng and bitter bean seeds, but there are not many studies on the mechanism of action for the treatment of cryptosporidiosis. This study describes the mechanism of potential targets and pathways of action of oxymatrine for the treatment of cryptosporidiosis using network pharmacology and experimental verification, providing a research basis for future therapeutic studies of oxymatrine.

Our research group's previous study found that oxymatrine has a therapeutic effect on *Cryptosporidium parvus*^17^. After successful modeling, the average number of fecal oocysts in mice infected with *Cryptosporidium parvus* gradually increased and reached the highest on day 7. After treatment with oxymatrine, the average number of fecal oocysts in infected mice began to decrease on day 5. After 2 weeks of treatment, the infection intensity approached grade 0. Meanwhile, histopathological results showed that the intestinal villi of the mice infected with *Cryptosporidium parvus* were basically repaired after oxymatrine treatment, and the structure tended to be complete and the arrangement tended to be orderly. The height of villus increased and the depth of crypt decreased, which proved that oxymatrine can promote the repair of damaged intestinal mucosa in mice, so as to achieve the purpose of treating cryptosporidiosis.

First, we performed a target database search to obtain a total of 47 intersecting targets of oxymatrine for the treatment of cryptosporidiosis, including the core targets of ALB, IL-6, TNF, AKT1, CASP3, and SRC. Subsequently, enrichment and analysis of these targets yielded a total of 324 GO functional entries and 84 KEGG pathways. The GO functional enrichment analysis showed that biological processes included negative regulation of cysteine-type endopeptidase activity involved in the apoptotic process, positive regulation of sequence-specific DNA binding transcription factor activity, collagen catabolic process, etc. Studies have shown that the KEGG pathway primarily functions via the TNF signaling pathway, apoptosis, IL-17 signaling pathway, NF-κB signaling pathway, etc. The TNF signaling pathway is one of the most important pathways in the human body. This pathway is mainly responsible for the body's inflammatory response. TNF is also known as TNF-α, a protein that plays a crucial role in the systemic inflammatory response, cell death, and the acute phase response. TNF-α binds to two types of receptors, i.e., TNFR1 and TNFR2. TNFR1 is expressed in most tissues. TNF-α binds to TNFR1 to form a complex that activates various signaling pathways. These pathways include the MARK signaling pathway, the NF-κB signaling pathway, and the apoptosis pathway. The first two signaling pathways trigger inflammation and release inflammatory factors such as IL-1, IL-6, and GM-CSF^22^. In order to investigate the mechanism of oxymatrine in treating cryptosporidiosis, we constructed a target-KEGG network and identified the top 5 involved genes, namely RELA, AKT1, TNF, CASP3, and IL-6. Based on the analysis, we identified several genes that shared commonalities with respect to previous cross-targets, namely RELA, AKT1, TNF, CASP3, and IL-6. In the process of molecular docking between oxymatrine and core targets, it was found that the binding energy of oxymatrine with AKT1, RELA, IL-6, CASP3, and TNF was less than − 5 kJ/mol, predominantly through hydrogen bonding. In addition, the binding sites were found, proving that oxymatrine has better binding properties with the above targets.Among them, RELA, TNF, and IL-6 are known to be involved in the TNF signaling pathway. Therefore, we decided to examine the possible mechanism of oxymatrine therapy for cryptosporidiosis by identifying the TNF/NF-κB signaling pathway.

Upon binding to TNFR1, TNF-α activates various proteases, leading to IκBα degradation and subsequent release of NF-κB. Released NF-κB enters the cell nucleus, regulating pathological processes such as cellular inflammation. It can reduce the severity of inflammation by inhibiting the activity of TNF-α and blocking the NF-kB signaling pathway^23^. Studies have shown that *Cryptosporidium* can cause the increase of TNF-α and IL-6 after infection of dairy cows, causing inflammation^24^. At the same time, TNF-α has been found as the central mediator of inflammation to induce the secretion of IL-6, IL-1 and other inflammatory factors, causing the inflammatory response after *Cryptosporidium* infection in mice. It plays an important role in the immune response of the host infected with *Cryptosporidium*^25^. This plays a vital role in the host's immune response to the infection^26^. *Cryptosporidium* infection in neonatal mice increases the release of TNF-α from inflammatory monocytes, leading to a rapid and significant increase in FITC-Dextran permeability. This indicates that *Cryptosporidium* infection changes intestinal permeability through the production of TNF-α by inflammatory factors^27^. For the anti-inflammatory effects of oxymatrine, there is also evidence that oxymatrine achieves anti-inflammation by down-regulating IFN-γ, TNF-α, IL-2, IL-6, IL-17A and IL-1β^28^. In addition, in the study of Miaohua Liu et al.^29^, they found that oxymatrine exerted anti-inflammatory effects by regulating the release of inflammatory mediators such as TNF-α from inflammatory cells and inhibiting the TLR4/NF-κB signaling pathway. Although oxymatrine is effective in anti-inflammatory aspects, the efficacy varies between individuals. Some people occasionally have mild nausea, bloating, headache, dizziness and other adverse reactions, but disappear a few days later or when stopping the drug use. Studies have shown that oxymatrine has neurotoxic effects, and one of its main toxic target organs is the nervous system. It can suppress the central nervous system of mice and impair their balance and coordination^30^. Therefore, further research is needed prior to clinically using this drug in the treatment of cryptosporidiosis. In this study, based on the results of network pharmacology, we established a mouse model of cryptosporidiosis, validated the TNF/NF-κB signaling pathway, and revealed the inflammatory response of cryptosporidiosis on the host and the anti-inflammatory effect of oxymatrine. The experimental results showed that the expression of TNF-α, NF-κB, and IL-6 in the intestinal mucosal tissues of mice in the infected group was significantly higher compared with the control group. This suggests that the infection of *Cryptosporidium* microsporidium causes the overexpression of inflammatory factors, thus aggravating the inflammatory injury. However, the expression of TNF-α, NF-κB and IL-6 was reversed after treated with oxymatrine, suggesting that oxymatrine can achieve the therapeutic effect of cryptosporidiosis through the TNF/NF-κB signaling pathway.

## Conclusion

In summary, this study used network pharmacology and experimental validation to investigate the mechanism of action of oxymatrine in the treatment of cryptosporidiosis. The results revealed that oxymatrine exerts its therapeutic effects through the synergistic effect of multiple core targets and signaling pathways. In vivo experiments preliminarily verified that oxymatrine can control intestinal inflammation by regulating the TNF/NF-κB signaling pathway and down-regulating the expression of TNF-α, NF-κB, and IL-6, thereby contributing to the treatment of cryptosporidiosis. This study underscores that oxymatrine's therapeutic benefits in cryptosporidiosis involve multiple targets through multiple pathways and molecular functions, which lays a solid foundation for further in-depth exploration of the mechanism of its action and paves the way for related experimental studies.

### Supplementary Information

Supplementary Information 1.

Supplementary Information 2.

## Data Availability

The datasets analyzed during the current study are available in the Traditional Chinese Medicine Systems Pharmacology Database (TCMSP, https://tcmsp-e.com/tcmsp.php),PharmMapper database (http://www.lilab-ecust.cn/pharmmapper/),Uniprot database (https://www.uniprot.org/),Gene Cards Database, Online Mendelian Inheritance in Man (OMIM, https://www.genecards.org/),Therapeutic Target Database (TTD, https://db.idrblab.net/ttd/), Comparative Toxicogenomics Database (CTD, http://ctdbase.org/),Database for Annotation Visualization and Integrated Discovery (DAVID, https://david.ncifcrf.gov/),Protein Data Bank (PDB, https://www.rcsb.org/),PubChem database (https://pubchem.ncbi.nlm.nih.gov/).
