# Supplementary material for: Mechanism of oxymatrine in the treatment of cryptosporidiosis through TNF/NF-κB signaling pathway based on network pharmacology and experimental validation
Source: Sci Rep. 2024 Jun 24;14:14469. doi: 10.1038/s41598-024-65362-0 (PMC11196726; doi:10.1038/s41598-024-65362-0)

Mechanism of oxymatrine in the treatment of cryptosporidiosis through TNF/NF-κB signaling pathway based on network pharmacology and experimental validation

Xiaoning Zhang[1 #], Jie Shi[1 #], Yilong Lu[1#], Rui Ji[3*], Zhiyu Guan[1*], Fujun Peng[1], Chunzhen Zhao[2], Wei Gao[1], Feng Gao[2]

[1]College of Basic Medical Sciences, Shandong Second Medical University, Weifang,China

[2]College of Pharmacy, Shandong Second Medical University, Weifang,China

[3]College of Traditional Chinese Medicine, Shandong Second Medical University, Weifang ,China

[#] These authors contributed equally:Xiaoning Zhang, Jie Shi, Yilong Lu: xiaoningzhang2022@163.com, 17763352001@163.com, 2297802842@qq.com.

[*] Corresponding Author: Rui Ji, College of Traditional Chinese Medicine, Shandong Second Medical University, Weifang ,China,261053, Email address: jirui2012@126.com

Zhiyu Guan, College of Basic Medical Sciences, Shandong Second Medical University, Weifang,China,261053,Email address: braveguan_001@163.com

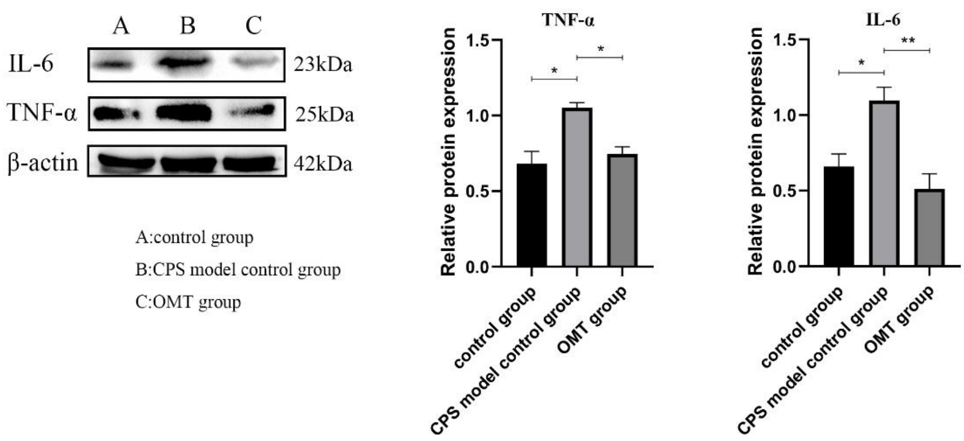

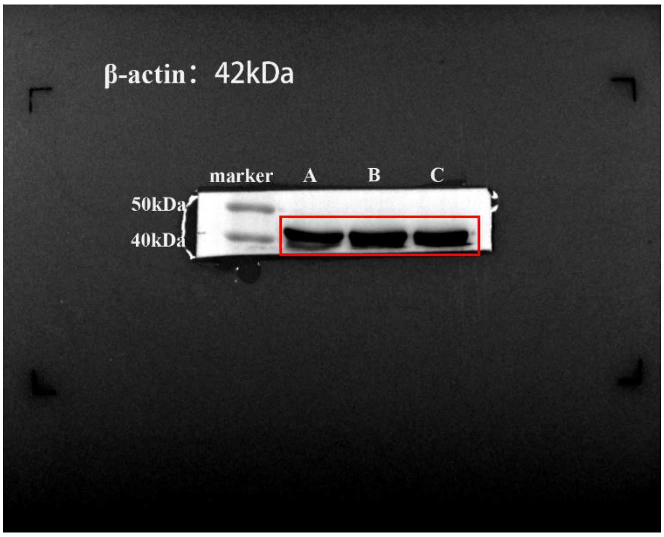

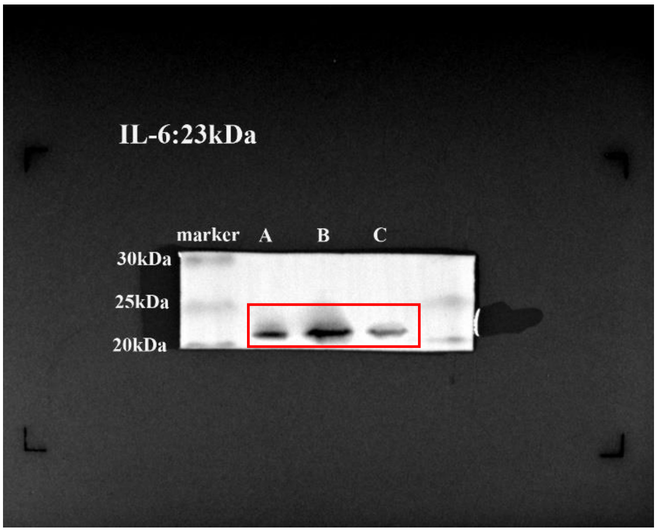

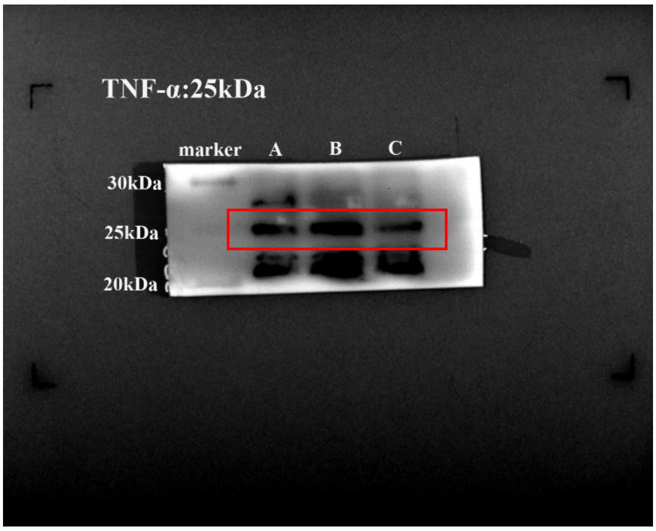

The red box indicates the area of the original blots used in the manuscript.

The following are the repeated experimental groups.

Second time:

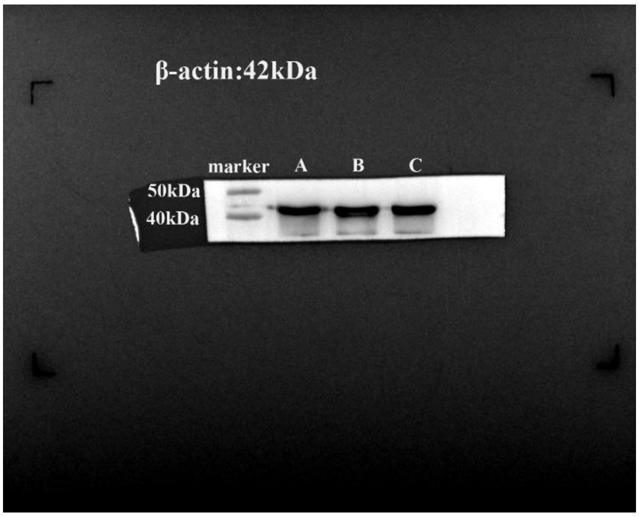

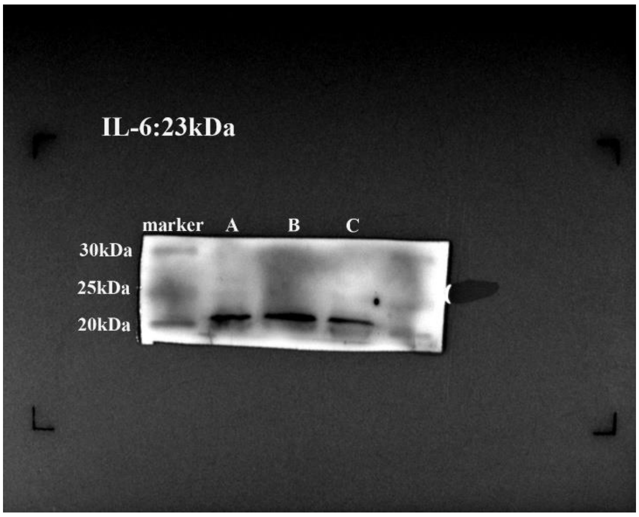

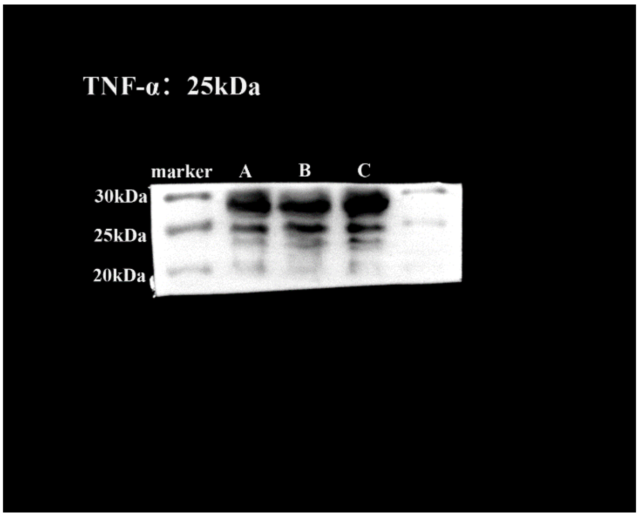

The third time:

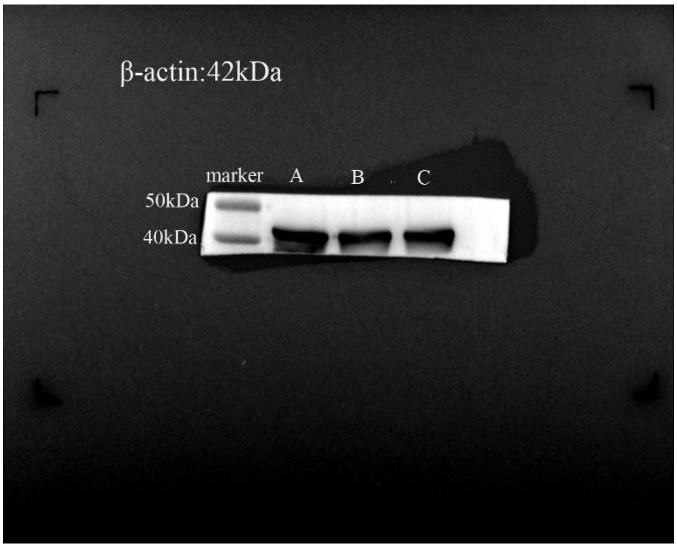

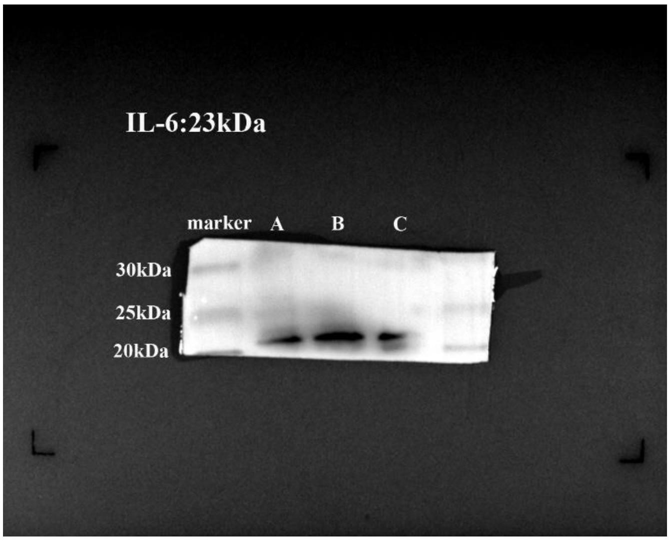

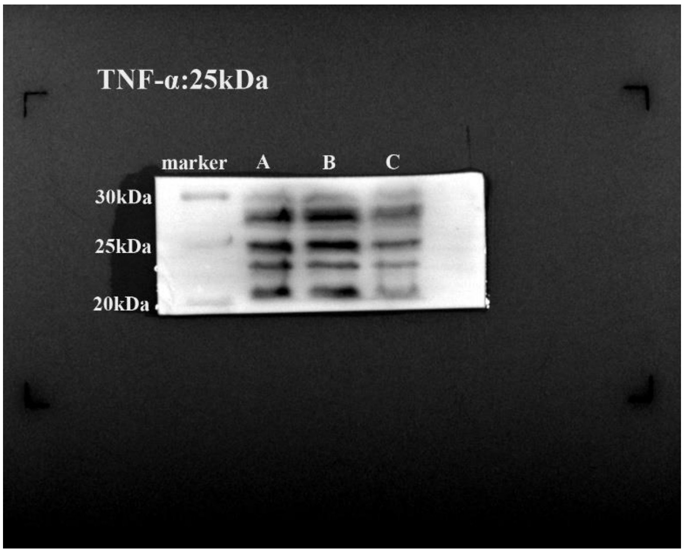

Supplement: Supplementary file 2 — Supplementary Information 2. [file 41598_2024_65362_MOESM2_ESM.pdf]
